# CCR2 inhibition sequesters multiple subsets of leukocytes in the bone marrow

**DOI:** 10.1038/srep11664

**Published:** 2015-07-24

**Authors:** Naoki Fujimura, Baohui Xu, Jackson Dalman, Hongping Deng, Kohji Aoyama, Ronald L Dalman

**Affiliations:** 1 Departments of Surgery, Stanford University School of Medicine, Stanford, CA 94305, USA; 2 Department of Vascular Surgery, Saiseikai Central Hospital, Minato-Ku Mita 1-4-17, Tokyo 108-0073, Japan; 3 Department of Hygiene and Health Promotion Medicine, Kagoshima University School of Medicine, Sakuragaoka 8-35-1, Kagoshima 890-0075, Japan

## Abstract

Chemokine receptor CCR2 mediates monocyte mobilization from the bone marrow (BM) and subsequent migration into target tissues. The degree to which CCR2 is differentially expressed in leukocyte subsets, and the contribution of CCR2 to these leukocyte mobilization from the BM are poorly understood. Using red fluorescence protein CCR2 reporter mice, we found heterogeneity in CCR2 expression among leukocyte subsets in varying tissues. CCR2 was highly expressed by inflammatory monocytes, dendritic cells, plasmacytoid dendritic cells and NK cells in all tissues. Unexpectedly, more than 60% of neutrophils expressed CCR2, albeit at low levels. CCR2 expression in T cells, B cells and NK T cells was greatest in the BM compared to other tissues. Genetic CCR2 deficiency markedly sequestered all leukocyte subsets in the BM, with reciprocal reduction noted in the peripheral blood and spleen. CCR2 inhibition via treatment with CCR2 signaling inhibitor propagermanium produced similar effects. Propagermanium also mitigated lipopolysaccharide-induced BM leukocyte egress. Consistent with its functional significance, CCR2 antibody staining revealed surface CCR2 expression within a subset of BM neutrophils. These results demonstrate the central role CCR2 plays in mediating leukocyte mobilization from the BM, and suggest a role for CCR2 inhibition in managing monocytes/macrophages-mediated chronic inflammatory conditions.

By migrating from their producing organ(s) through the bloodstream, secondary lymphoid and peripheral target tissues, leukocytes play a central role in immune surveillance, inflammation, and response to injury. The process of migration is tightly controlled by expression of endothelial adhesion molecules and chemoattractants (primarily chemokines) in target tissues, as well as receptor expression on the leukocytes themselves^1^,^2^. For example, expression of the peripheral node addressin in secondary lymphoid tissues, and L-selectin on lymphocytes, mediates lymphocyte migration into peripheral lymph nodes (LNs), bronchus-associated lymphoid tissues and areas of inflammation^3^,^4^,^5^. Alternatively, mucosal cell adhesion molecule-1 and the α4β7 integrin mediate lymphocyte migration into Peyer’s patches, intestinal and pancreatic LNs^3^,^4^,^6^. Chemokine receptor CCR7 and its ligands CCL19 and CCL21 mediate lymphocyte migration into LNs, Peyer’s patches, inflamed pancreatic islets and the egress of lymphocytes from peripheral tissues into draining LNs^2^,^7^,^8^,^9^.

The chemokine receptor CCR2 is expressed mainly by inflammatory monocytes, and binds two ligands, CCL2 and CCL7^10^,^11^,^12^,^13^. A large body of existing research has established the role that CCR2 and its ligands play in recruiting inflammatory monocytes into target tissues, where they differentiate into proinflammatory and anti-inflammatory macrophages and promote tissue injury and remodeling, respectively^14^. CCR2 and its ligands are also critically important in mobilizing monocytes from the bone marrow (BM), the monocyte-generating organ, into the bloodstream under both physiological and pathological conditions^15^,^16^,^17^,^18^,^19^. CCR2 has thus been recognized as an attractive potential target inhibition in the treatment of macrophage-mediated chronic diseases such as atherosclerosis.

Propagermanium (PG), an organic germanium compound, is approved for treating patients with chronic hepatitis type B in Japan^20^. PG exerts immunomodulatory effects by interacting with glycosylphosphatidylinositol-anchored proteins associated with CCR2, interrupting CCR2-mediated signaling and chemotaxis without disrupting the receptor itself or its ligands^21^. PG has proven effective in suppressing a range of chronic inflammatory conditions primarily mediated by inflammatory monocytes and macrophages, such as experimental atherosclerosis, ischemia-induced brain injury and fibrosis^22^,^23^,^24^,^25^,^26^,^27^. The degree to which CCR2 is expressed by leukocytes other than monocytes, however, and the role that CCR2 may play in mobilization of these leukocytes from the BM, have not been comprehensively investigated to date.

In this study, we used red fluorescent protein (RFP) CCR2 reporter mice^28^ to evaluate the frequency and intensity of CCR2 expression on individual leukocyte subsets. Further, we determined the degree to which genetic deletion, or pharmacological inhibition of CCR2 with PG, alters the relative and absolute numbers of individual leukocyte subsets in the BM, peripheral blood, spleen and LNs under resting conditions. Finally, we examined the influence of PG on lipopolysaccharide (LPS)-stimulated BM leukocyte mobilization. Our results provide additional insight into the process of CCR2-mediated mobilization of BM leukocytes under both physiological and pathological conditions, further underscoring the potential role receptor inhibition may play in ameliorating chronic inflammatory conditions.

## Results

### Differential expression patterns of CCR2 in leukocyte subsets under resting conditions

**Figure 1:**
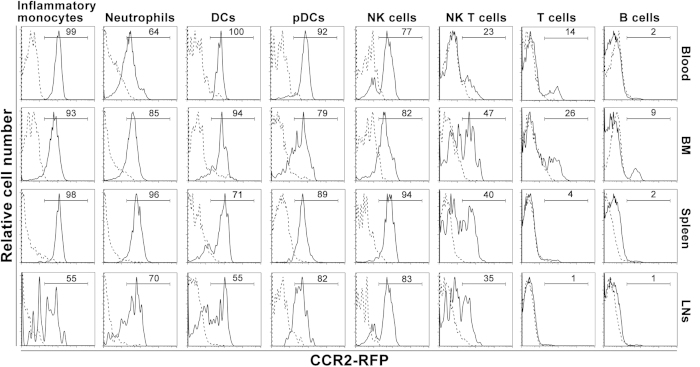
Representative flow cytometric histographs for CCR2 expression in leukocyte subsets as detected by CCR2 reporter protein RFP. Cell suspensions of the peripheral blood, BM, spleen and cervical LNs from the CCR2 RFP reporter mice (CCR2-RFP^+/−^) and age-and sex-matched non-transgenic WT C57BL/6J mice were stained with differential subset-specific mAbs. In flow cytometric analyses, subset identification was inflammatory monocytes (CD11b^+^Ly-6C^high^), neutrophils (CD11b^+^Ly-6G^+^), DCs (CD11c^+^PDCA-1^−^), pDCs (CD11c^+^PDCA-1^+^), T cells (CD3^+^B220^−^), B cells (B220^+^CD3^−^), NK T cells (CD3^+^DX5^+^), and NK cells (CD3^−^B220^−^DX5^+^). CCR2-expressing cells were defined as RFP-positive cells. In each graph, number represents the percentage of RFP-expressing CCR2-positive cells in a given subset. Solid and dashed lines indicate the CCR2 RFP protein expression in CCR2 RFP reporter mice and background autofluorescence staining in WT non-transgenic C57BL/6J mice, respectively.

**Figure 2:**
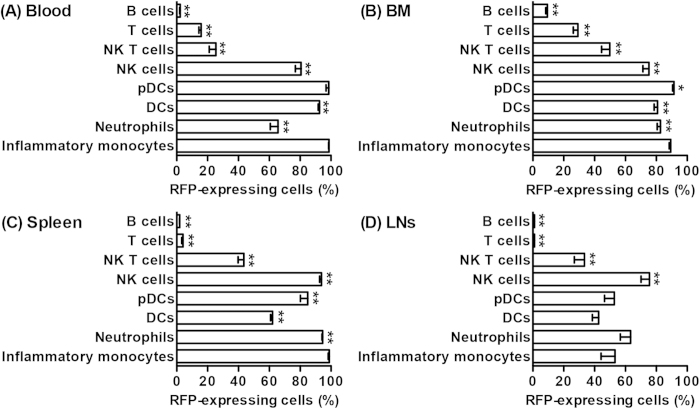
Heterogeneity of CCR2 expression frequency among subsets and tissues. Cell suspensions of the blood, BM, spleen and cervical LNs from CCR2-RFP^+/−^ CCR2 reporter mice were stained with subset-specific mAbs. CCR2-expressing cells were defined as the RFP-expressing cells in each subset. All data are mean and SD of the frequencies of CCR2-expressing cells in individual subsets obtained from 5 mice in each group. Welch’s unpaired T test, *p < 0.05 and **p < 0.01 compared to inflammatory monocytes in the same tissue.

Flow cytometry was performed on whole blood and cell suspensions derived from the BM, spleen and LNs of CCR2-RFP^+/−^ CCR2 reporter mice following staining with subset-specific monoclonal antibodies (mAbs). Age-and sex-matched non-transgenic C57BL/6J mice were used as the negative control (background autofluorescence). Supplementary Fig. 1 shows the flow cytometric gating strategies for identifying individual leukocyte subsets in all tissue compartments. In all experiments, inflammatory monocytes, neutrophils, dendritic cells (DCs), plasmacytoid DCs (pDCs), T cells, B cells and NK T cells were defined as CD11b^+^Ly-6C^high^, CD11b^+^Ly-6G^+^, CD11c^+^PDCA-1^−^, CD11c^+^PDCA-1^+^, CD3^+^B220^−^, CD3^−^B220^+^ and CD3^+^DX5^+^ cells, respectively, in total leukocytes as gated by forward and side scatters. NK cells were defined as DX5^+^ cells in CD3^−^B220^−^ cells. As demonstrated in Figs 1 and 2, with the exception of blood neutrophils (65%) and splenic DCs (62%), CCR2 was expressed by greater than 80% of myeloid cells present in the blood, BM and spleen. In LNs, however, CCR2 expression across all leukocyte subsets was generally less prevalent. In all tissues tested, greater than 75% of NK cells expressed CCR2, with the highest frequency noted in the spleen. NK T cells expressed CCR2 at lower frequency (25 to 50%) than NK cells did. Approximately 29% of BM T cells, 15% of blood T cells, and 9% of BM B cells also expressed CCR2.

**Figure 3:**
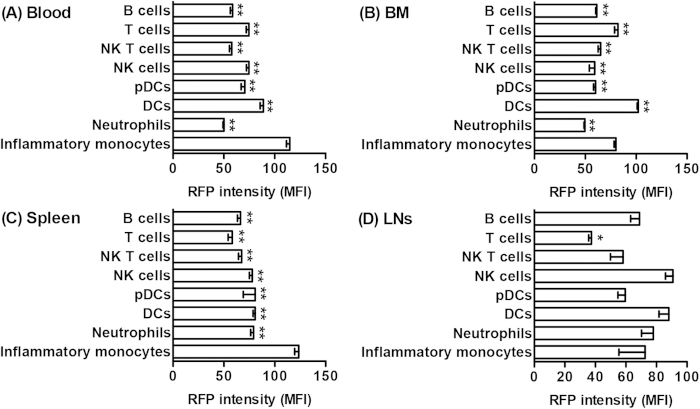
Heterogeneity of CCR2 expression levels among subsets and tissues. In CCR2-RFP^+/−^ CCR2 reporter mice, CCR2 expression levels in CCR2 (RFP)-expressing cells for individual subsets were measured by the mean fluorescence intensity (MFI) of RFP. All data are mean and SD of RFP MFI obtained from 5 mice in each group. Welch’s unpaired T test, *p < 0.05 and **p < 0.01 compared to inflammatory monocytes in a same tissue.

Next, we analyzed subset-specific CCR2 expression levels by measuring the mean fluorescence intensity (MFI) of RFP in CCR2 (RFP)-expressing cells. Consistent with the findings in the expression frequency, CCR2 expression levels varied among tissues and subsets (Fig. 3). The highest CCR2 levels (MFI: >100) were observed for inflammatory monocytes in the blood and spleen as well as DCs in the BM, with moderate levels (MFI: 70–100) for most other subsets. The lowest intensity was obtained from T cells in LNs (MFI: <70). These results indicate heterogeneous CCR2 expression among tissues and subsets, both in terms of the frequency of cells with receptor expression, as well as the intensity of expression in receptor-expressing cells.

### CCR2 deletion promotes BM leukocyte sequestration

To determine the degree to which CCR2 expression and activity influence the relative numbers of individual leukocyte subsets in the peripheral blood, BM, spleen and LNs, flow cytometric analysis was performed in CCR2-RFP^+/−^ (intact CCR2 expression) and CCR2-RFP^+/+^ (CCR2 deficiency) mice. As summarized in Supplementary Table 1, the relative numbers of inflammatory monocytes in all compartments were significantly lower in CCR2-RFP^+/+^ mice compared to CCR2-RFP^+/−^ mice, decreased by 84%, 72%, and 62% in the blood, spleen, and LNs, respectively. In CCR2-RFP^+/+^ mice, small but statistically significant decreases in the relative numbers of neutrophils were also noted in LNs and spleen, as well as DCs in the blood and LNs, and pDCs in LNs as compared to CCR2-RFP^+/−^ mice. Whereas NK cells in all tissues did not differ between two strains, NK T cells in the blood and spleen, and T cells in the blood and spleen were significantly more prevalent in CCR2-RFP^+/+^ mice than in CCR2-RFP^+/−^ mice. B cells were also more prevalent in BM but lower in the blood and spleen in CCR2-RFP^+/+^ mice than those in CCR2-RFP^+/−^ mice.

**Figure 4:**
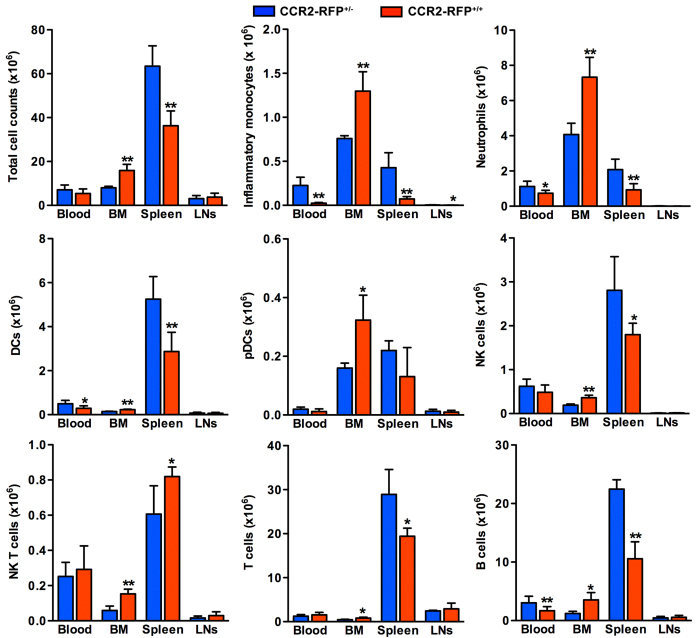
Influence of genetic CCR2 deficiency on leukocyte sequestration in the BM. Total leukocytes were prepared from the blood, BM, spleen and cervical LNs of CCR2-RFP^+/+^ (CCR2 deficiency) and CCR2-RFP^+/−^ mice (intact CCR2 expression), counted, and stained with subset-specific mAbs followed by flow cytometric analysis. Absolute numbers for individual subsets were calculated from total cell counts and subset frequency. All data are mean and SD of absolute cell counts obtained from 5 mice in each group. Welch’s unpaired T test, *p < 0.05 and *p *< 0.01 compared to CCR2-RFP^+/−^ mice.

Next, we analyzed the effects of CCR2 deficiency on the absolute numbers of specific leukocyte subsets (Fig. 4). In CCR2-RFP^+/+^ mice, total leukocyte counts in the spleen were reduced by 44%, with reciprocal increase in the BM by nearly two thirds as compared to CCR2-RFP^+/−^ mice. A small reduction in total blood leukocytes was also noted, but did not reach statistical significance. CCR2 deficiency was associated with greater than 40% reduction in all myeloid subsets and B cells, and a 90% reduction in inflammatory monocytes in the blood. In the spleen of CCR2-RFP^+/+^ mice, with the exception of NK T cells, all leukocyte subsets were significantly reduced. Correspondingly, cell counts for all BM leukocytes of CCR2-RFP^+/+^ mice were 1.6–2.9 times greater than those in CCR2-RFP^+/−^ mice. In LNs, except for inflammatory monocytes, there were no differences in subset cellularity between two strains. These results suggest that expression or activity of CCR2 influences egress of all subsets of leukocytes from the BM.

### Pharmacological CCR2 inhibition retains multiple subsets of leukocytes in the BM in steady state

To explore whether, and by what magnitude, pharmacological inhibition of CCR2 by PG recapitulates the phenotypic characteristics of CCR2 deficiency in regards to BM and circulating leukocyte populations, 10 week (wk) old male wild type (WT) C57BL/6J mice were treated with PG (50 mg/kg/day via oral gavage), or an equal volume of vehicle (phosphate-buffered saline, PBS), for 14 days. At sacrifice, body weight in PG-treated mice (102.5 ± 3.8% of baseline) was undistinguishable of that in vehicle-treated mice (100.5 ± 1.6% of baseline). PG treatment was not associated with excessive mortality or obvious morbidity.

**Figure 5:**
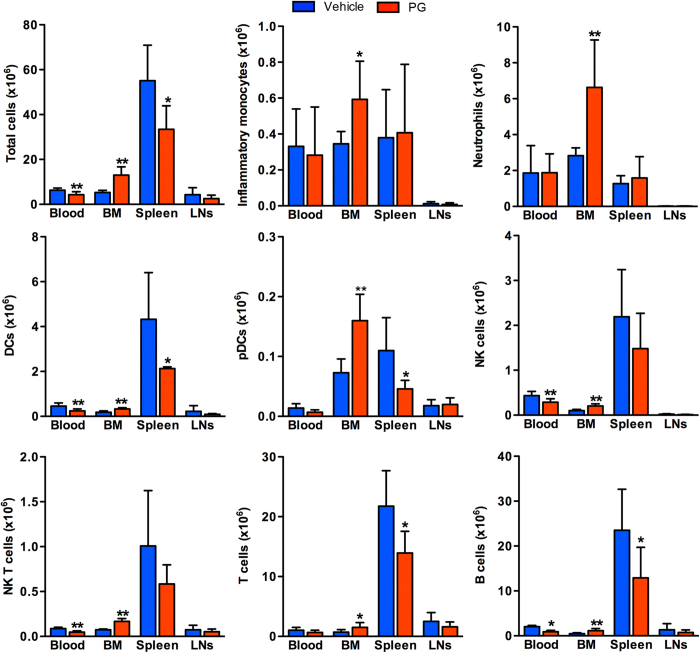
Influence of CCR2 inhibitor PG on leukocyte sequestration in the BM. Male C57BL/6J mice at 10 wks of age were daily treated with PG (50 mg/kg/day) or an equal volume of PBS (as the vehicle control) via oral gavage. Fourteen days following each treatment, mice were sacrificed, and total leukocyte suspensions were prepared from the blood, BM, spleen and cervical LNs, counted, stained with subset-specific mAbs followed by flow cytometric analysis. Absolute numbers for individual subsets were calculated from total cell counts and subset frequency. All data are expressed as mean and SD of absolute cell counts obtained from 7 mice in each group. Welch’s unpaired T test, *p < 0.05 and *p *< 0.01 compared to vehicle treatment.

With the exception of BM inflammatory monocytes (decreased), the relative numbers of leukocyte subsets in all compartments was not significantly affected by PG-treatment (Supplementary Table 2). PG did, however, reduce total leukocyte counts in the blood (32%) and spleen (40%), with a reciprocal 1.5 fold increase in the BM (Fig. 5). Significant differences between two groups were only seen for DCs, NK cells, NK T cells and B cells in the blood. PG treatment also significantly reduced the absolute numbers of DCs (50%), pDCs (59%) and T (34%) and B cells (45%) in the spleen. No similar influence was noted in LNs. Importantly, as noted in the deficient mice (CCR2-RFP^+/+^), substantial sequestration of all leukocyte subsets was noted in the BM, reaching 1.7–2.4 times of those noted in vehicle-treated mice. Thus pharmacological inhibition partially replicates the phenotype characteristic of CCR2 deficient mice in term of leukocyte compartmentalization. These findings reinforce the significance of CCR2 expression and activity on egress of all leukocyte subsets from BM under resting conditions.

### PG treatment attenuates LPS-dependent leukocyte egress from the BM

Peripheral monocytosis is characteristic of varying pathologic conditions including systemic infection and atherosclerosis^14^. Monocytosis, or mobilization of monocytes from the BM, is induced in part by Toll-like receptor (TLR)-stimulated CCL2 expression on mesenchymal stromal cells^18^. LPS treatment increases circulating inflammatory monocytes, with reciprocal decreases noted in the BM^18^. To assess the influence of PG on egress of leukocytes from the BM in response to pathological stimuli, PG was administered 2 hours (h) before, and immediately following LPS injection. Four hours thereafter, the relative and absolute numbers of leukocytes in the various compartments were analyzed via flow cytometry. As seen in Supplementary Table 3, except for increased relative number of splenic DCs following PG treatment, the relative numbers of the other subsets in the remaining compartments did not differ significantly between the two groups. Trends were noted for decreased relative numbers of inflammatory monocytes and B cells in the blood and spleen, neutrophils in the BM, and NK T cells in the BM and LNs in PG-treated mice. Slight, though not significant, increases in relative numbers of circulating NK T cells, BM B cells, and splenic pDCs were also noted in PG-treated mice.

**Figure 6:**
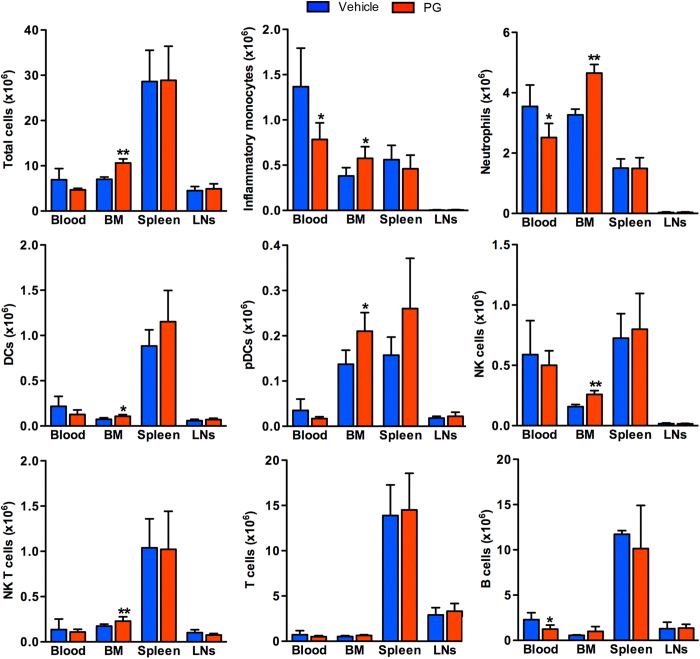
PG treatment attenuates LPS-stimulated leukocyte egress from the BM. Male C57BL/6J mice at 10 wks of age were treated with PG (50 mg/kg) or PBS (as the vehicle control) 2 h prior to and immediately following intraperitoneal injection of 20 ng LPS. Four hours thereafter, mice were sacrificed, and leukocytes were prepared from the blood, BM, spleen and cervical LNs. Subset frequency was determined by single cell suspension immunofluorescence staining and flow cytometric analysis. Absolute numbers for individual subsets were calculated from total cell counts and subset frequency. All data are mean and SD. Welch’s unpaired T test, *p < 0.05 and **p < 0.01 compared to vehicle treatment. n = 5 mice in each group.

Figure 6 summarizes the effects of PG treatment on the absolute numbers of individual leukocyte subsets in LPS-injected mice. PG treatment was associated with reduced total circulating leukocytes, and a significant increase in BM leukocytes in LPS-injected mice. No influence was noted in LNs or the spleens. All circulating subsets were reduced from 15% to 52%. Significance was reached, however, only for inflammatory monocytes, neutrophils and B cells. Reciprocally, leukocyte counts in all subsets increased in the BM of PG-treated mice, ranging from 22% to 78%. PG treatment did not affect subset cellularity in LNs or the spleen. Thus PG treatment does offset, to a significant extent, pathologically stimulated egress of leukocytes from the BM.

### Differential expression of surface and intracellular CCR2 in neutrophils

**Figure 7:**
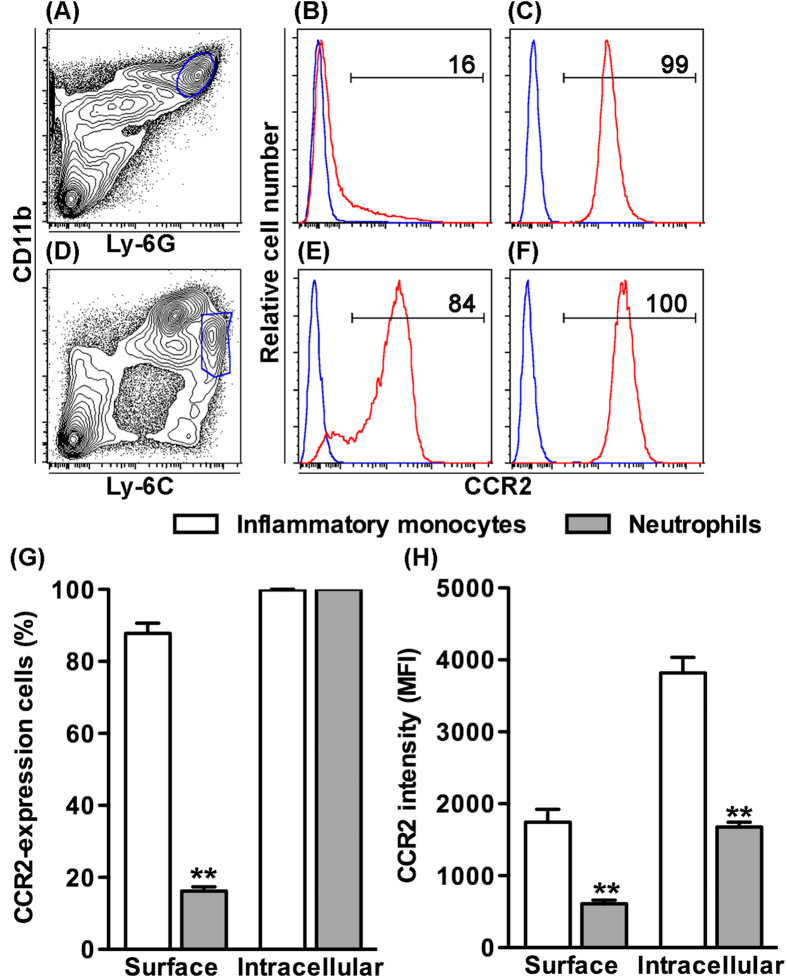
BM neutrophils express surface and intracellular CCR2. BM leukocyte suspensions were differentially stained for surface and intracellular CCR2 expression with mAbs against leukocyte subset markers (CD11b, Ly-6C and Ly-6G) and CCR2 (or its isotype control mAb), and analyzed by flow cytometry. (**A–F**) Representative flow cytometric plots for the surface (**B**,**E**) and intracellular (**C**,**F**) CCR2 staining in gated CD11b^+^Ly-6G^+^ neutrophils (**A**) and CD11b^+^Ly-6C^high^ inflammatory monocytes (**D**). Blue and red lines in B, C, E and F indicate the staining with CCR2 and its isotype control mAbs, respectively. Number in each plot is the percentage of CCR2-expressing cells in neutrophils (**B**,**E**) and inflammatory monocytes (**C**,**F**). **(G)** Mean and SD of the percentages of neutrophils and inflammatory monocytes expressing surface or intracellular CCR2. **(H)** Mean and SD of the CCR2 expression intensity, as measured by the MFI of CCR2 mAb staining, in CCR2-expressing neutrophils and inflammatory monocytes. Welch’s unpaired T test, **p < 0.01 compared to inflammatory monocytes. n = 5 mice in each group.

As described previously, CCR2 expression was detected in more than 80% of BM neutrophils in CCR2-RFP^+/−^ reporter mice, and genetic and pharmacological CCR2 inhibition prompted BM neutrophil sequestration. To determine whether neutrophils express surface and/or intracellular CCR2, we used an anti-CCR2 mAb to stain the surface and intracellular expression of CCR2 in BM neutrophils as compared to inflammatory monocytes (Fig. 7). Surface CCR2 expression was detected on 16% of neutrophils (16.2 ± 1.2%), which was significantly lower than the percentage noted on inflammatory monocytes (87.8 ± 2.8%). The expression levels on BM neutrophils (610.2 ± 51.5), as measured by MFI, were approximately a third of that on inflammatory monocytes (1745.5 ± 179.5). For intracellular staining, all neutrophils (99.9 ± 0.1%) and inflammatory monocytes (100.0 ± 0.0%) expressed CCR2. Similar to surface staining, intracellular CCR2 expression levels (1676.8 ± 65.1) were significantly lower in neutrophils than those in inflammatory monocytes (3818.5 ± 216.2). These results indicate that CCR2 is expressed on the surface of a subset of neutrophils and thus may mediate neutrophil egress from the BM.

## Discussion

We report the novel use of a murine RFP construct to quantify leukocyte CCR2 expression and its influences on leukocyte subsets in peripheral blood and varying immune organs. Both genetic deficiency of CCR2, and pharmacological inhibition in WT mice were associated with sequestration of multiple leukocyte linages within the BM, with reciprocal reductions noted in circulating and splenic leukocytes under resting conditions. PG also limited leukocyte egress from the BM in response to LPS injection in WT mice. Based on these results, CCR2 expression and activity appears to play an important role in regulating leukocyte egress, migration and peripheral distribution.

Consistent with prior studies^10^,^13^, almost all inflammatory monocytes in the CCR2-RFP^+/−^ reporter mice expressed CCR2. More pDCs (50–98%) and NK cells (75–93%) expressed CCR2 than has been reported previously using antibody-based characterization studies^29^,^30^, however, this prevalence was consistent with that reported by studies employing fluorescent CCR2 ligand to localize and quantify functional CCR2^29^. Similar to previous reports^13^,^14^,^29^,^31^,^32^, RFP technology confirmed CCR2 expression on nearly all NK cells but few T (<30%) and B cells (<10%), more in the BM than in other compartments. These results confirm that leukocyte CCR2 expression varies widely, depending on cell types and tissues, under resting conditions.

CCR2 is also expressed by neutrophils in response to stimuli such as bacterial components or growth factors, but not classically under resting conditions^33^,^34^,^35^,^36^,^37^,^38^,^39^. RFP technology demonstrated CCR2 expression on all splenic neutrophils, and greater than 60% of remaining neutrophils in other compartments under physiological (unstimulated) conditions. Further, as demonstrated in BM cells using CCR2 antibody staining, neutrophils expressed both intracellular and surface CCR2, although significantly less in the latter. These results agree with the prior finding that myeloid peroxidase and Gr-1-expressing neutrophils, differentiated from mouse myeloid progenitors by G-CSF, expressed CCR2 and migrated towards CCL2 in *in vitro* transwell migration assays^40^. Thus, CCR2-positive neutrophils detected via CCR2-RFP^+/−^ reporter mice contain a subset of surface CCR2-expressing cells detectable via CCR2 antibody. Additionally, the high sensitivity of reporter RFP may also enable detection of additional neutrophils expressing surface CCR2 below threshold detectable by antibody staining.

Myeloid cells, including inflammatory monocytes, neutrophils, DCs and pDCs, are constitutively mobilized from the BM into the bloodstream under resting conditions. The findings of BM retention and reduced circulating myeloid cell populations in receptor deficient or pharmacologically inhibited mice derived from these experiments generally agree with prior studies emphasizing the importance of CCR2 activity in modulating monocyte mobilization from the BM^15^,^16^,^17^,^18^,^19^. Deficiency of transcription factor Runx2 also results in down-regulation of CCR2 and CCR5 on pDCs, impaired the migration towards CCL2 and CCL5, and pDC retention in the BM^30^. However, the current study is the first to suggest that CCR2 also influences egress of DCs or neutrophils from the BM, in addition to inflammatory monocytes and pDCs^15^,^16^,^17^,^18^,^19^. Migration of circulating neutrophils into areas of inflammation or injury is recognized to be mediated in part via CCR2/CCL2 signaling, or was accelerated by the presence of CCR2-expressing monocytes^33^,^34^,^35^,^36^,^37^,^38^,^39^. In the present study, approximately 80% and 16% of total and surface CCR2, respectively, was detected on BM neutrophils via CCR2-RFP^+/−^ reporter technology and CCR2 antibody staining. CCR2-expressing monocytes and CCL2 are present under resting conditions in the BM^18^. Thus, neutrophil egress could proceed in a CCR2 and/or monocyte-dependent manner. Additionally, B cell CCR2 expression negatively regulates the CXCL12/CXCR4 cascade critical for BM retention of hematopoietic cells^32^,^41^. Crosstalk between the CCR2 and CXCR4 signaling pathways may also contribute to additional myeloid cell egress from the BM.

Additional mechanisms are recognized to regulate the emigration of B cells and NK cells from the BM. CXCR4/CXCL12 and sphingosine -1-phosphate (S1P)/S1P receptor 1 activity affect B cell egress, whereas S1P/S1P receptor 5 signaling mediates NK cell egress^41^,^42^,^43^,^44^,^45^,^46^. In the present study, both genetic and pharmacological inhibition of CCR2 signaling reduced BM egress and circulating levels of B cells and NK cells. These results reinforce emerging evidence of a role for CCR2 signaling in regulating egress of these non-myeloid cells from the BM. In prior studies, more CCR2-deficient NK cells accumulated in the BM of chimeric mice than WT NK cells following BM cell transplantation^47^. Additionally, CCL2 was able to recruit circulating NK cells to injured limbus and corneal stroma^48^. Given the recognized expression of CCL2 in the BM^18^, CCR2/CCL2 may influence NK cell egress as well.

No prior evidence has suggested a role for CCR2 in the mobilization of B cells from BM. Because CCR2-expressing B cells disrupt downstream signaling of CXCR4^32^, ubiquitously expressed on hematopoietic cells and critical for BM cell retention^41^,^44^, CCR2 may contribute to B cell egress by interfering with the CXCR4 signaling cascade. Additionally, CCR2 may potentially mediate B cell egress by modulating S1P receptor 1^42^,^46^, although this remains to be clarified.

BM T cells are predominantly “antigen-experienced”, as indicated by high expression of CD44 (CD4 T cells) or CD122 (CD8 T cells), while BM NK T cells represent more a mixed population, recirculating through the bloodstream or produced or expanded *in situ*^49^. The S1P/S1P1 pathway has been previously recognized to mediate T cell egress from the BM^50^,^51^. Mice deficient in S1P1 also have fewer NK T cells in the bloodstream, imputing a role for S1P1 in regulating egress of NK T cells^52^. In the present study, both T and NK T cell subsets were significantly sequestered in the BM due to CCR2 deficiency or pharmacological inhibition. These results suggest that an alternative or additional mechanism to the canonical S1P/S1P1 pathway may exist for regulating egress of T and NK T cells from the BM, although further studies will be necessary to clarify this relationship.

In contrast to effects on the BM and peripheral blood, CCR2 inhibition or deficiency imparted negligible influence on LN immune cell cellularity under resting conditions. These findings are consistent with the current recognized role of CCR7, and its ligands CCL19 and CCL21 expressed on LN venules and stromal cells, in mediating lymphocyte migration to LNs^2^. In the spleen, absence of CCR2 activity caused reduction in the populations of most leukocyte subsets, although these did not all reach statistical significance. Changes in the splenic inflammatory cell populations may result from altered inward migration, egress or retention within the spleen.

In prior published studies, monocyte egress from the spleen has been shown to be dependent on angiotensin II type 1 receptor signaling^53^. No role has been previously recognized for CCR2 signaling in mediating migration of circulating monocytes into the spleen^15^. The decreases in splenic leukocyte cellularity demonstrated in our experiments may simply represent an indirect consequence of massive BM sequestration resulting from CCR2 deficiency or inhibition. Paradoxically, CCR2 deficiency increased, whereas PG treatment reduced, splenic NK T cells. Based on the available results, there is no specific explanation for this finding, other than the possibility of a lineage-specific, unrecognized genetic compensation for embryonic CCR2 ablation.

Monocyte egress from the BM is augmented in pathological conditions such as bacterial infection and chronic diseases^15^,^16^,^17^,^18^,^19^. LPS enhances BM monocyte mobilization by stimulating CCL2 production by BM stromal cells^18^. Thus, the suppression of LPS-accelerated BM leukocyte mobilization (including monocytes) by PG treatment may result in part from impaired CCL2/CCR2 interaction, rather than downstream affects on the CCR2 receptor signaling system itself. Further, PG treatment altered neither LN nor splenic leukocyte cellularity in LPS-injected mice. These differential effects imply that, unlike in the setting of genetic deficiency, pharmacological CCR2 inhibition may not be sufficient to dampening adaptive immunity in the spleen and organ-draining LNs, respectively.

In conclusion, these results suggest that CCR2 expression or activity regulates egress of multiple leukocyte subsets from the BM. PG or alternative CCR2 inhibitors may offer novel opportunity for treating chronic conditions mediated by BM-derived leukocyte, particularly monocyte, mobilization.

## Methods

### Mice

WT C57BL/6J mice and CCR2-RFP^+/+^ (CCR2 deficiency) mice on C57BL/6J genetic background were purchased from The Jackson Laboratory, Bar Harbor, ME. To generate littermate CCR2-RFP^+/−^ (CCR2 reporter mice with intact CCR2 expression) and CCR2-RFP^+/+^ (CCR2 deficiency) mice for experiments, male CCR2-RFP^+/+^ mice were mated with female C57BL/6J mice. Resulting female CCR2-RFP^+/−^ mice were further backcrossed with male CCR2-RFP^+/+^ mice, and offspring was screened for the presence of CCR2 and RFP using anti-CCR2 mAb fluorescence staining and flow cytometry. Animals used in all experiments were male at 10 wks of age. All animal experiments were carried out in accordance with the Stanford Laboratory Animal Care Guidelines and Regulation and approved by the Stanford Administrative Panel on Laboratory Animal Care.

### MAbs and other reagents

MAbs used in this study were PE/Cy7-CD11b (M1/70), Alexa Fluor®488-Ly-6C (HK1.4), PE-Ly-6G (1A8) and PE-CD11c (N418) (BioLegend, San Diego, CA); Alexa Fluor®647-PDCA-1 (129c) and FITC-B220 (RA3-6B2) (eBioscience, San Diego, CA); PE-Pan-NK cells (DX5, BD Biosciences, San Jose, CA); Alexa Fluor®647-CD3 (HM3421, Caltag Laboratories, Burlingame, CA); and APC-CCR2 (475301) and its isotype control (141945) (R&D Systems Inc, Minneapolis, MN). PG (SEROCION®) and LPS were purchased from Sanwa Kagaku Kenkyusho Co, Ltd, Nagoya, Japan and Sigma-Aldrich Co, LLC, St. Louis, MO, respectively.

### Pharmaceutical inhibition of CCR2 with PG

To investigate the effects of PG treatment on leukocyte subsets under resting conditions, PG was administered for 14 days via oral gavage. Previous rodent studies have demonstrated efficacy of PG at 5–500 mg/kg/day in suppressing varying pathologies^23^,^54^, we thus chose 50 mg/kg/day as a relatively low dose within the effective range to minimize toxicity. Because of its short-half life (2.5 h), freshly prepared PG in PBS was administered q8 h for a daily dose of 50 mg/kg. In the vehicle control group, an equal volume of PBS was administered using the same regimen. Body weight, morbidity and mortality were monitored daily. To investigate the effects of PG treatment following LPS treatment, mice were preconditioned with PG (50 mg/kg) to ensure sufficient drug levels. Two hours later, mice were simultaneously treated with 20 ng LPS via intraperitoneal injection, and 50 mg/kg PG via oral gavage or an equal volume of PBS as vehicle control^18^. Four hours thereafter, mice were euthanized by carbon dioxide inhalation. Whole blood, BM, spleens and cervical LNs were harvested for cell isolation and subsequent cell suspension immunofluorescence staining.

### Flow cytometric analysis

Mice were anesthetized by inhaling isoflurane, and tail vein blood was obtained for total leukocyte counting (1:20 dilution in 3% acetic acid solution) and whole blood immunofluorescence staining (5 mM EDTA in PBS). At the sacrifice, left femur bone, spleen and cervical LNs were excised. Leukocytes were isolated from BM plugs, spleen and LNs using mechanical dissociation. After lysing erythrocytes with the lysing buffer (150 mM NH_4_CI, 10 mM KHCO and 0.1 mM Na_2_EDTA, pH 7.2) and PBS washing, cells were suspended in RMPI-1640 medium and counted using a hemocytometer. Cell suspensions were stained with differentially fluorochrome-conjugated mAb combinations: CD3/B220/PanNK mAbs for T cells (CD3^+^B220^−^), B cells (CD3^−^B220^+^), NK cells (CD3^−^B220^−^DX5^+^) and NK T cells (CD3^+^DX5^+^); CD11b/Ly-6C/Ly-6G mAbs for inflammatory monocytes (CD11b^+^Ly-6C^high^) and neutrophils (CD11b^+^Ly-6G^+^); and CD11c/PDCA-1 mAbs for DCs (CD11c^+^PDCA-1^−^) and pDCs (CD11c^+^PDCA-1^+^). Strategies for flow cytometric identification of individual leukocyte subsets in all tissues were detailed in Supplementary Figure 1. All staining were performed on ice for 30 min in PBS followed by extensive PBS washes to remove residual mAbs^3^,^4^. Staining patterns were acquired using a BD FACS Calibur flow cytometer with CellQuest software (BD Biosciences) and analyzed using FlowJo software (Ver 9.6.4, Tree Star Inc, Eugene, OR). The frequency and levels of CCR2 expression were determined by staining leukocytes from individual tissues of CCR2-RFP^+/−^ CCR2 reporter mice. RFP-positive cells were defined as CCR2-expressing cells, and its expression frequency was given as the percentage of RFP-positive cells in each subset. For individual subsets, CCR2 expression levels in CCR2 (RFP)-expressing cells were estimated as the MFI of RFP. Additionally, the absolute cell numbers of individual subsets were calculated by multiplying total leukocyte number and subset frequency in all experiments.

Additionally, CCR2 antibody staining was performed to determine surface and intracellular CCR2 expression on BM neutrophils and inflammatory monocytes. To detect surface CCR2, leukocyte suspensions were simultaneously stained with differential fluorescence dye-labeled mAb against CD11b, Ly-6C, Ly-6G and CCR2 (or its isotype control). To detect intracellular CCR2, leukocytes were stained with mAbs against CD11b, Ly-6C and Ly-6G followed by extensive PBS washes. The cells were then fixed with Biolegend intracellular staining fix buffer, permeabilized with Biolegend permeabilization washing buffers, and stained with anti-CCR2 or its isotype control mAb according to the manufactures’ instruction. Staining patterns were acquired as described above. The percentages of neutrophils and inflammatory monocytes expressing surface or intracellular CCR2, as well as the expression intensity in CCR2-positive cells were calculated using FlowJo software.

### Statistical analyses

All data were presented as mean and standard deviation (SD). Welch’s unpaired T test was used to test the statistical difference between two groups. P < 0.05 was considered to be significant.

## Additional Information

**How to cite this article**: Fujimura, N. *et al.* CCR2 inhibition sequesters multiple subsets of leukocytes in the bone marrow. *Sci. Rep.* **5**, 11664; doi: 10.1038/srep11664 (2015).

## Supplementary Material

Supplementary Information
