# Supplementary material for: CCR2 inhibition sequesters multiple subsets of leukocytes in the bone marrow
Source: Sci Rep. 2015 Jul 24;5:11664. doi: 10.1038/srep11664 (PMC4513281; doi:10.1038/srep11664)
Supplement: Supplementary Information [file srep11664-s1.pdf]

Title:
CCR2 inhibition sequesters multiple subsets of leukocytes in the bone marrow

Authors:
Naoki Fujimura[1,2], Baohui Xu[1*], Jackson Dalman[1], Hongping Deng[1], Kohji Aoyama[3] & Ronald L Dalman[1]
[1]Departments of Surgery, Stanford University School of Medicine, Stanford, CA 94305, USA
[2]Department of Vascular Surgery, Saiseikai Central Hospital, Minato-Ku Mita 1-4-17, Tokyo 108-0073, Japan
[3]Department of Hygiene and Health Promotion Medicine, Kagoshima University School of Medicine, Sakuragaoka 8-35-1, Kagoshima 890-0075, Japan

***Correspondence to:**
Baohui Xu, MD/PhD
Department of Surgery
Stanford University School of Medicine
Room P323, MSLS Building
1201 Welch Road, Stanford, CA 94305
Phone: 650-724-7582
Email: baohuixu@stanford.edu

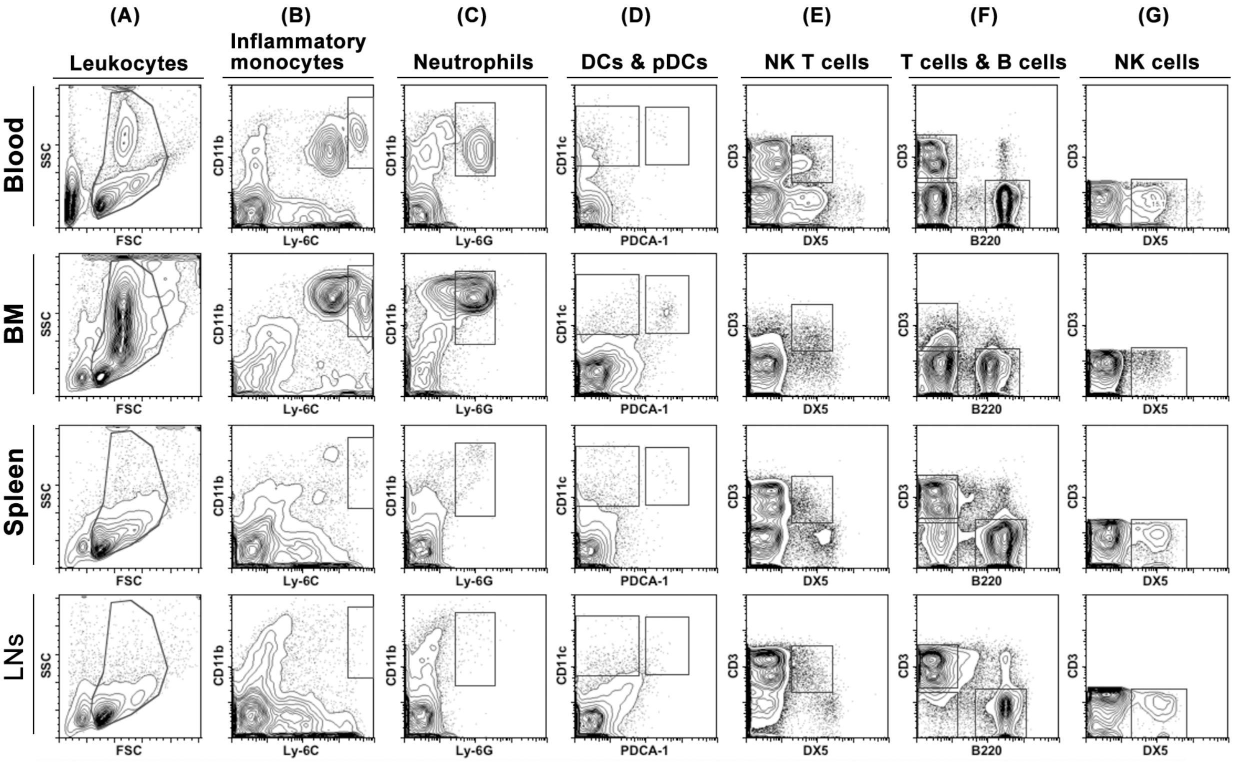

Supplementary Figure 1. Flow cytometric strategies for identifying leukocyte subsets. In all experiments, tail vein blood (in 5 mM EDTA/PBS) and cell suspensions isolated from femur bone, spleen and cervical LNs were stained with differentially fluorescence dye-labeled mAb combinations. (A): FSC and SSC identified total leukocytes for further analyses in panels B-F. (B): CD11b and Ly-6C mAb staining identified inflammatory monocytes as CD11b^{+}Ly-6C^{high} cells. (C): CD11b and Ly-6G mAb staining identified neutrophils as CD11b^{+}Ly-6G^{+} cells. (D): CD11c and PDCA-1 mAb staining identified DCs and pDCs as CD11c^{+}PDCA-1^{-} and CD11c^{+}PDCA-1^{+} cells, respectively. (E, F): CD3, B220 and DX5 mAb staining identified NK T cells as CD3^{+}DX5^{+} cells (E), T cells as CD3^{+}B220^{-} cells (F), B cells as CD3^{-}B220^{+} cells (F), and CD3^{-}B220^{-} cells (F). (G): DX5 mAb staining identified NK cells as DX5^{+} cells in CD3^{-}B220^{-} cells gated in panel F.

Supplementary Table 1. Effects of CCR2 deficiency on the relative numbers of individual leukocyte subsets in the peripheral blood and lymphoid tissues

Tissue	Blood		BM		LNs		Spleen	
	CCR2-RFP$^{+/-}$	CCR2-RFP$^{+/+}$	CCR2-RFP$^{+/-}$	CCR2-RFP$^{+/+}$	CCR2-RFP$^{+/-}$	CCR2-RFP$^{+/+}$	CCR2-RFP$^{+/-}$	CCR2-RFP$^{+/+}$
Inflammatory monocytes	3.1 ± 0.3	0.5 ± 0.2**	9.6 ± 0.7	8.3 ± 0.6*	0.13 ± 0.03	0.05 ± 0.03**	0.7 ± 0.2	0.2 ± 0.1**
Neutrophils	15.9 ± 2.4	14.9 ± 4.5	51.2 ± 5.4	46.8 ± 3.5	0.4 ± 0.1	0.2 ± 0.1*	3.2 ± 0.5	2.5 ± 0.5**
DCs	7.0 ± 0.4	5.4 ± 0.4**	1.8 ± 0.2	1.5 ± 0.2	2.2 ± 0.2	1.7 ± 0.2**	8.2 ± 0.5	7.8 ± 0.9
pDCs	0.3 ± 0.0	0.2 ± 0.1	2.0 ± 0.2	2.0 ± 0.2	0.4 ± 0.1	0.2 ± 0.1*	0.4 ± 0.1	0.3 ± 0.2
NK cells	9.0 ± 1.3	9.1 ± 0.7	2.4 ± 0.4	2.3 ± 0.2	0.4 ± 0.1	0.4 ± 0.1	4.4 ± 0.6	5.0 ± 0.4
NK T cells	3.5 ± 0.3	5.3 ± 0.6**	0.8 ± 0.3	1.0 ± 0.1	0.5 ± 0.1	0.7 ± 0.2	1.0 ± 0.1	2.3 ± 0.3**
T cells	17.5 ± 1.1	28.8 ± 0.8**	5.5 ± 1.6	5.1 ± 0.7	78.6 ± 2.1	78.6 ± 5.1	45.4 ± 2.9	54.2 ± 4.8*
B cells	42.1 ± 2.9	30.6 ± 3.2**	15.4 ± 4.8	21.7 ± 4.8	14.3 ± 2.1	14.2 ± 4.5	35.8 ± 3.5	28.8 ± 3.0*

All data are given as the percentages of individual leukocyte subsets in total leukocytes. CCR2-RFP$^{+/-}$: CCR2 intact mice. CCR2-RFP$^{+/+}$: CCR2 deficient mice. Welch's unpaired T test, *$P<0.05$ & **$P<0.01$ compared to WT mice. n=5 mice in each group.

Supplementary Table 2. Effects of pharmacological CCR2 inhibitor PG treatment on the relative numbers of individual leukocyte subsets in the peripheral blood and lymphoid tissues

Tissue	Blood		BM		LNs		Spleen	
	Vehicle	PG	Vehicle	PG	Vehicle	PG	Vehicle	PG
Inflammatory monocytes	5.5 ± 3.8	6.1 ± 4.8	6.6 ± 1.5	4.6 ± 1.0*	0.2 ± 0.1	0.2 ± 0.2	0.8 ± 0.6	1.5 ± 1.5
Neutrophils	28.7 ± 20.1	40.8 ± 14.7	53.5 ± 3.9	50.0 ± 6.4	0.4 ± 0.2	0.4 ± 0.3	2.5 ± 1.2	5.5 ± 4.7
DCs	7.2 ± 1.8	6.1 ± 2.1	3.4 ± 0.8	2.7 ± 0.8	5.4 ± 2.4	4.2 ± 2.1	7.3 ± 1.9	6.1 ± 1.7
pDCs	0.2 ± 0.1	0.2 ± 0.1	1.4 ± 0.3	1.3 ± 0.4	0.6 ± 0.4	1.0 ± 0.7	0.2 ± 0.1	0.1 ± 0.0
NK cells	7.1 ± 2.0	6.9 ± 1.2	2.0 ± 0.5	1.7 ± 0.6	0.7 ± 0.2	0.6 ± 0.2	3.9 ± 1.5	4.2 ± 1.4
NK T cells	1.4 ± 0.1	1.2 ± 0.5	1.4 ± 0.2	1.3 ± 0.4	2.1 ± 1.1	2.4 ± 1.4	1.8 ± 0.7	1.8 ± 0.7
T cells	17.0 ± 7.7	17.4 ± 10.0	13.5 ± 7.1	13.5 ± 9.0	65.6 ± 11.4	64.8 ± 7.9	40.2 ± 7.0	43.1 ± 8.0
B cells	33.2 ± 16.5	23.2 ± 11.3	9.1 ± 3.3	9.5 ± 4.1	27.3 ± 8.3	28.4 ± 5.6	41.3 ± 7.5	36.4 ± 9.3

All data are given as the percentages of individual leukocyte subsets in total leukocytes. Welch's unpaired T test, *P<0.05 compared to vehicle-treated mice. n=7 mice in each group.

Supplementary Table 3. Effects of pharmacological CCR2 inhibitor PG treatment on the relative numbers of individual leukocyte subsets in the peripheral blood and lymphoid tissues in LPS-treated mice

Tissue	Blood		BM		LNs		Spleen	
	Vehicle	PG	Vehicle	PG	Vehicle	PG	Vehicle	PG
Inflammatory monocytes	20.2 ± 3.1	16.6 ± 3.2	5.4 ± 0.9	5.3 ± 0.9	0.1 ± 0.1	0.1 ± 0.1	2.0 ± 0.7	1.7 ± 0.6
Neutrophils	53.8 ± 7.9	53.4 ± 7.5	47.1 ± 2.0	43.4 ± 2.9	0.7 ± 0.5	0.8 ± 0.4	5.3 ± 0.7	5.3 ± 0.9
DCs	3.2 ± 1.2	2.7 ± 0.3	1.1 ± 0.2	1.0 ± 0.2	1.4 ± 0.4	1.4 ± 0.3	3.1 ± 0.4	4.0 ± 0.2**
pDCs	0.5 ± 0.2	0.4 ± 0.1	2.0 ± 0.3	2.0 ± 0.2	0.4 ± 0.1	0.5 ± 0.2	0.6 ± 0.2	0.9 ± 0.2
NK cells	8.4 ± 2.1	10.6 ± 2.1	2.3 ± 0.2	2.5 ± 0.4	0.4 ± 0.1	0.3 ± 0.1	2.6 ± 0.6	2.7 ± 0.7
NK T cells	1.8 ± 0.8	2.3 ± 0.6	2.5 ± 0.2	2.1 ± 0.3	2.3 ± 0.7	1.6 ± 0.4	3.8 ± 1.3	3.4 ± 0.7
T cells	10.0 ± 2.4	10.9 ± 2.4	7.5 ± 0.9	6.0 ± 0.5*	64.3 ± 5.6	67.7 ± 4.9	48.9 ± 3.6	50.0 ± 2.0
B cells	36.0 ± 15.1	26.8 ± 11.3	7.9 ± 0.6	9.3 ± 4.7	28.1 ± 9.1	28.0 ± 6.8	40.7 ± 6.6	33.9 ± 9.9

All data are given as the percentages of individual leukocyte subsets in total leukocytes. Welch's unpaired T test, *P<0.05 & **P<0.01 compared to vehicle-treated mice. n=5 mice in each group.
